# Prediction of incident atrial fibrillation in community-based electronic health records: a systematic review with meta-analysis

**DOI:** 10.1136/heartjnl-2021-320036

**Published:** 2021-10-04

**Authors:** Ramesh Nadarajah, Eman Alsaeed, Ben Hurdus, Suleman Aktaa, David Hogg, Matthew G D Bates, Campbel Cowan, Jianhua Wu, Chris P Gale

**Affiliations:** 1 Leeds Institute of Data Analytics, University of Leeds, Leeds, UK; 2 Department of Cardiology, Leeds Teaching Hospitals NHS Trust, Leeds, UK; 3 School of Computing, University of Leeds, Leeds, UK; 4 Department of Cardiology, South Tees Hospitals NHS Foundation Trust, Middlesbrough, UK; 5 School of Dentistry, University of Leeds, Leeds, Leeds, UK

**Keywords:** atrial fibrillation, primary care, electronic health records, meta-analysis

## Abstract

**Objective:**

Atrial fibrillation (AF) is common and is associated with an increased risk of stroke. We aimed to systematically review and meta-analyse multivariable prediction models derived and/or validated in electronic health records (EHRs) and/or administrative claims databases for the prediction of incident AF in the community.

**Methods:**

Ovid Medline and Ovid Embase were searched for records from inception to 23 March 2021. Measures of discrimination were extracted and pooled by Bayesian meta-analysis, with heterogeneity assessed through a 95% prediction interval (PI). Risk of bias was assessed using Prediction model Risk Of Bias ASsessment Tool and certainty in effect estimates by Grading of Recommendations, Assessment, Development and Evaluation.

**Results:**

Eleven studies met inclusion criteria, describing nine prediction models, with four eligible for meta-analysis including 9 289 959 patients. The CHADS (Congestive heart failure, Hypertension, Age>75, Diabetes mellitus, prior Stroke or transient ischemic attack) (summary c-statistic 0.674; 95% CI 0.610 to 0.732; 95% PI 0.526–0.815), CHA_2_DS_2_-VASc (Congestive heart failure, Hypertension, Age>75 (2 points), Stroke/transient ischemic attack/thromboembolism (2 points), Vascular disease, Age 65–74, Sex category) (summary c-statistic 0.679; 95% CI 0.620 to 0.736; 95% PI 0.531–0.811) and HATCH (Hypertension, Age, stroke or Transient ischemic attack, Chronic obstructive pulmonary disease, Heart failure) (summary c-statistic 0.669; 95% CI 0.600 to 0.732; 95% PI 0.513–0.803) models resulted in a c-statistic with a statistically significant 95% PI and moderate discriminative performance. No model met eligibility for inclusion in meta-analysis if studies at high risk of bias were excluded and certainty of effect estimates was ‘low’. Models derived by machine learning demonstrated strong discriminative performance, but lacked rigorous external validation.

**Conclusions:**

Models externally validated for prediction of incident AF in community-based EHR demonstrate moderate predictive ability and high risk of bias. Novel methods may provide stronger discriminative performance.

**Systematic review registration:**

PROSPERO CRD42021245093.

## Introduction

Atrial fibrillation (AF) is the most common sustained cardiac arrhythmia and is associated with a five-fold increased risk of stroke.^1 2^ This risk can be reduced by two-thirds by a number of effective oral anticoagulants,^3 4^ but it is estimated that 30% of patients living with AF are undiagnosed and its first manifestation is stroke in more than 10% of patients.^5 6^

International guidelines recommend opportunistic rather than systematic screening in asymptomatic patients, using age over 65 years as the only risk predictor.^2 7^ In many European countries, a large proportion of the population is registered in primary care with a routinely collected electronic health record (EHR).^8 9^ A multivariable prediction model that uses this data source to give a more discriminative assessment of risk could allow far-reaching, cost-effective targeted screening.

There are several prediction models for incident AF in community-dwelling individuals but they have predominantly been tested in prospective cohorts and their performance may not translate to EHR data.^10^ To show utility for targeting screening in the general population using real-world EHR, a model would need to have been tested in EHR or administrative claims databases relevant to the general population or primary care (herein referred to as community-based EHR).

We performed a systematic review and meta-analysis with a number of aims. First, to identify prediction models for incident AF derived or validated in community-based EHR. Second, to summarise the performance of individual prediction models to understand if any would be suitable for use in targeted screening. Third, to summarise the methods by which prediction models have been developed in EHR to inform future research within the field.

## Methods

We reported this systematic review and meta-analysis in accordance with the Preferred Reporting Items for Systematic Reviews and Meta-Analyses guidelines (online supplemental material).^11^

10.1136/heartjnl-2021-320036.supp1 Supplementary data

### Search strategy and inclusion criteria

The research question was framed using the CHecklist for critical Appraisal and data extraction for systematic Reviews of prediction Modelling Studies (CHARMS) (online supplemental material).^12^ We searched the Medline and Embase databases through the Ovid platform from inception through 23 March 2021. We used a combination of keywords and subject headings related to AF, prediction models and EHR based on previous literature.^13–15^ The search was limited to the English language and to human studies. The full search strategy is provided in online supplemental material. We manually searched the reference lists of included studies and previous systematic reviews.^13 14^ Duplicates were removed using Endnote’s duplicate identification strategy and then manually.

To be eligible for inclusion a study had to:

1. Be an original study in human adults (≥18 years of age).
2. Develop and/or validate a prediction model(s) for incident AF or atrial flutter (AFl) based on multivariable analysis in a community-based EHR. We included AFl as a co-outcome because it has a similar indication for anticoagulation.^2^
3. Be written in English.

Articles were excluded if they:

1. Included patients with AF or AFl at baseline.
2. Only reported measures of association between risk factors and incident AF rather than a full prediction model.
3. Studied only a subset of the general population, for example, individuals diagnosed with a particular morbidity.
4. Incorporated variables that would not be routinely available in community-based EHR (eg, ECG parameters) (online supplemental material).

We uploaded records to a systematic review web application (Rayyan, Qatar Computing Research Institute).^16^ Four investigators (RN, EA, BH and SA) independently screened them for inclusion by title, abstract, full text and supplementary materials. Disagreements were resolved by consultation with a fifth investigator (JW).

### Data extraction and quality assessment

Two investigators (RN and EA) independently extracted the data from the included studies based on CHARMS. This included the following domains: data source, participants, outcome(s), candidate predictors, sample size, missing data, and model development, performance and evaluation. Discrepancies were resolved with a third investigator (JW). All data came from the primary reference, unless otherwise stated.

To allow quantitative synthesis of the predictive performance of the models we extracted measures of discrimination and calibration.^17^ Discrimination quantifies the model’s ability to distinguish between individuals developing or not developing the outcome. We extracted data on the *c*-statistic (*c*-statistic=1 if the model discriminates perfectly, *c*-statistic=0.5 if discrimination no better than chance) or area under the receiver operating characteristic (AUROC) and corresponding 95% confidence interval (95% CI). When the 95% CI was not reported we calculated it using methods described by *Debray et al*.^17^ Calibration refers to the model’s accuracy of predicted probabilities; we extracted data on the p value of a goodness-of-fit test and the reported ratio for observed to expected events or calibration slope.

Two investigators (RN and JW) assessed each model in each study for risk of bias and applicability to our review question using the Prediction model Risk Of Bias ASsessment Tool (PROBAST).^18^ Discrepancies were resolved with a third investigator (CPG). Each model was assessed for risk of bias as either ‘high’, ‘unclear’ or ‘low’ in four domains (participants, predictors, outcomes and analysis) through a range of signalling questions. Applicability to our review question was assessed for each model in three domains (participants, predictors and outcomes) using the same scale.^18^

### Data synthesis and statistical analysis

We reported continuous variables as means±SD and categorical variables as percentages. Calibration was infrequently reported, so we restricted meta-analysis to discrimination through a summary measure of *c*-statistic and corresponding 95% CI. In our primary analysis we assessed overall discrimination for models that had ≥3 EHR cohorts with *c*-statistic data. When multiple *c*-statistic data for a model were reported in a single cohort by different studies we only included the first published study.

We calculated the 95% prediction interval (PI) to depict the extent of between-study heterogeneity and to indicate a possible range for prediction model performance in a new validation.^19^ When the 95% CI or PI of the summary *c*-statistic included 0.5 we concluded that there was insufficient evidence that the prediction model has statistically significant discriminatory ability.^13 20^ We used a Bayesian approach throughout as frequentist methods, where there are fewer studies or a mixture of study sizes, have produced PIs with poor coverage.^19^ The prior distributions specified are summarised in online supplemental material. A logit transformation was applied to the *c-*statistic prior to meta-analysis, as the between-study distribution of the *c-*statistic is often skewed.^21^ We conducted meta-analyses in R using the metafor and metamisc package (R foundation for Statistical Computing V.3.6.3).^22–24^

We performed a number of sensitivity analyses:

1. To only include studies where the participants’ domain in PROBAST assessment was ‘low’ or ‘unclear’ risk of bias.
2. To only include studies where the overall PROBAST assessment was ‘low’ or ‘unclear’ risk of bias.
3. Where a cohort had been reported multiple times we replaced the meta-analysis data with the data on the same cohort from any later study.
4. We excluded data from one of the Korean National Health Insurance Service Health screening cohort (NHIS-HEALS) and Korean National Health Insurance Service-based National Sample cohort (NHIS-NSC) because they originated from the same EHR database.

The Grading of Recommendations, Assessment, Development and Evaluation approach was used to assess the certainty of the evidence.^25^ The certainty of the evidence was graded as ‘high’, ‘moderate’, ‘low’ or ‘very low’. One investigator (RN) rated the certainty of the evidence for the primary outcome and this was checked by a second investigator (JW). The criteria used are summarised in online supplemental material.

### Patient and public involvement

Patients or the public were not involved in the design, conduct, reporting or dissemination plans of our research.

## Results

### Study selection

**Figure 1:**
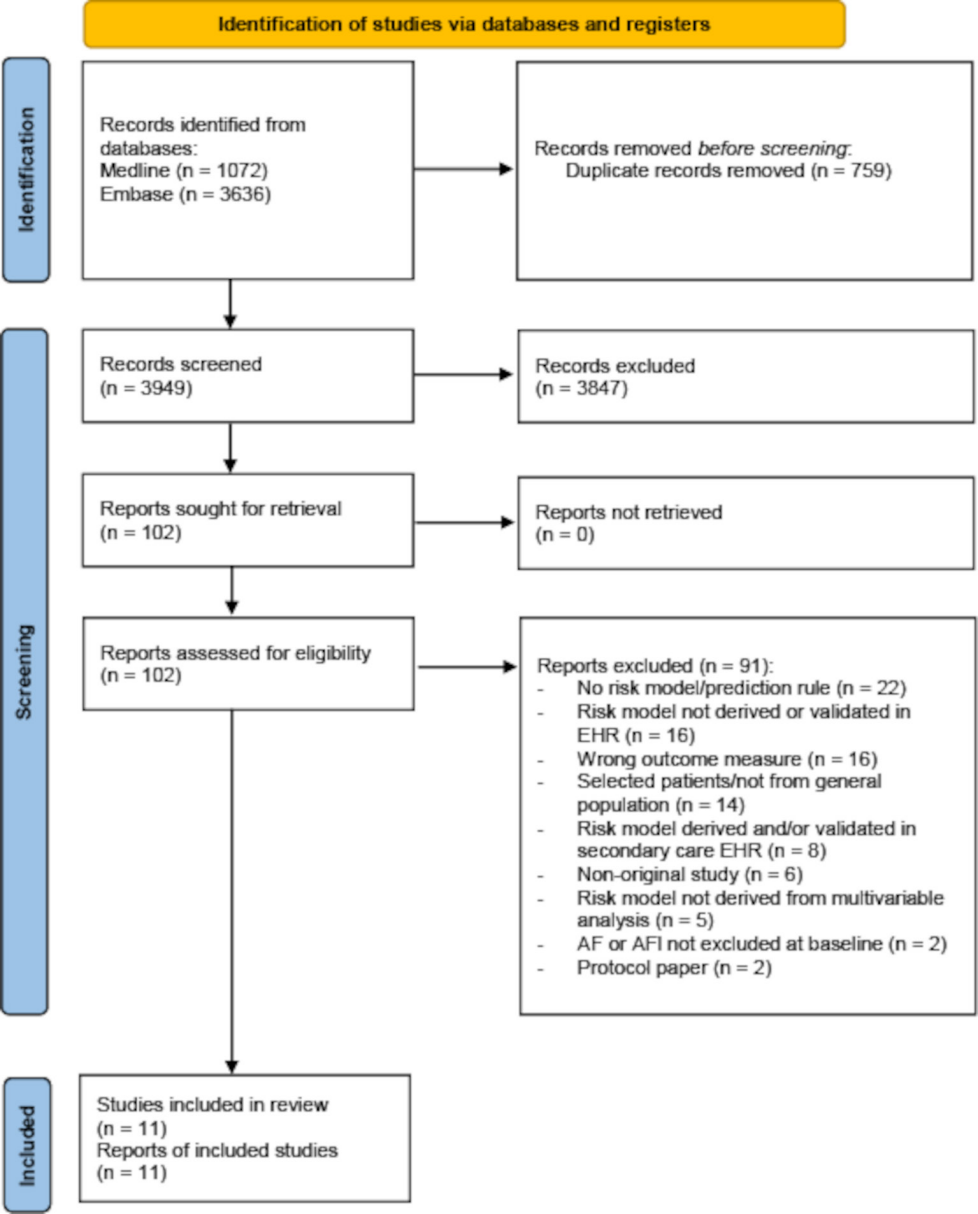
Flow diagram of literature search. AF, atrial fibrillation; AFl, atrial flutter; EHR, electronic health record.

The study selection process is described in figure 1. We identified 3949 unique records, reviewed 102 full-text reports and included 11 studies. A list of excluded studies that met a number of the inclusion criteria is available in online supplemental material.

### Characteristics of included studies

The 11 included studies were based on nine cohorts from eight EHR databases, located in Asia Pacific (n=3), Europe (n=3) and the Middle East (n=2) (table 1).^9 26–35^ The number of times a prediction model had been derived or validated in EHR was skewed to Asia Pacific (n=17) compared with Europe (n=5) and the Middle East (n=3) (table 2).

**Table 1:** Characteristics of included studies

Study	Cohort (country)	Study aim	EHR description	AF cases (n)/total patients (n) (%)	Age (mean±SD)	Female (%)	BMI (mean±SD)	Diabetes (%)	Hypertension (%)	Heart failure (%)	Outcome definition	Outcome coding	Enrolment period (mean F/U in years)	Exclusion criteria
Aronson *et al* 26	MHS (IL)	D	Ambulatory clinics	5660/96 778 (5.80)	62.0 ±9.0	53.7	28.2 ±5.1	13.5	34.3	1.00	AF, AFI	ICD codes	2005–2015 (10.0)	Past history of AF, incomplete follow-up
Chao *et al* 27	NHIRD (TW)	EV	National health insurance	9187/702 502 (1.30)	41.3 ±16.4	49.1	N/A	3.1	5.2	0.40	AF*	ICD codes*	2000–2009 (9.0)	Age <18 years, past history of cardiac arrhythmia, rheumatic heart disease
Hill *et al* 28	CPRD (UK)	D, EV	Nationwide primary care	95 607/2994 837 (3.19)	56.0 ±14.5	53.4	27.6 ±6.0	6.9	25.0	0.70	AF, AFI	Read codes	2006–2016 (N/A)	Age <30 years, past history of AF
Himmelreich *et al* 9	Nivel-PCD (NL)	EV	Nationwide primary care	5264/111 475 (4.72)	65.5 ±11.4	52.5	N/A	42.7	66.5	4.20	AF, AFl	ICPC-1 codes	2013–2018 (N/A)	Age <40 years, past history of AF
Hu W-S *et al*†^29^	NHIRD (TW)	D	National health insurance	14 212/682 237 (2.08)	41.3	49.3	N/A	2.1	15.1	0.80	AF	ICD codes	2000–2013 (7.1)	Age <18 years, past history of AF, incomplete data
Hu W-S and Lin C-L^30^	NHIRD (TW)	EV	National health insurance	12 051/692 691 (1.74)	41.3 ±16.3	49.4	N/A	N/A	14.4	0.49	AF	ICD codes	1996–2013 (10.9)	Age <18 years, past history of AF, incomplete data
Kim *et al*†^35^	NHIS-NSC (KR)	D, EV	National Health Insurance	5824/432 587 (1.35)	47.7	50.5	23.7	6.3	21.2	2.40	AF, AFl	ICD codes	2009–2013 (N/A)	Age <18 years, past history of AF, mitral valve stenosis or prosthetic valve disease, missing data for smoking or alcohol, change in residence
Li *et al*†^31^	YMID (CN)	D, EV	Regional medical insurance	921/471 446 (0.20)	47.0	47.3	N/A	4.0	9.7	0.15	AF	ICD codes	2001–2012 (4.1)	Past history of AF, incomplete data, readmission
	NHIS-HEALS (KR)	EV	National health examination programme	12 143/451 199 (2.69)	56.1 ±9.3	46.0	N/A	8.3	31.7	1.20	AF	ICD codes	2002–2013 (7.3)	Past history of AF, mitral stenosis, prosthetic heart valves, valve replacement or valvuloplasty or cardiomyopathy
Saliba *et al* 32	ClalitHS (IL)	EV	State-mandated health services	23 223/1062 073 (2.19)	65.7 ±11.2	54.7	N/A	25.3	48.9	4.30	AF	ICD codes	2012–2014 (2.9)	Age <50 years, past history of AF
Sekelj *et al* 33	Discover (UK)	EV	Regional primary care	17 880/604 135 (2.96)	52.2 ±13.3	51.0	27.0 ±6.1	23.2	17.9	0.50	AF, AFl	Read codes	2006–2013 (N/A)	Age <30 years, past history of AF, incomplete data for height, weight, BMI, systolic BP and diastolic BP
Suenari *et al* 34	NHIRD (TW)	EV	National health insurance	9174/670 804 (1.40)	42.4 ±16.0	49.1	N/A	3.2	5.5	0.40	AF	ICD codes	2000–2011 (9.0)	Age <20 years, past history of cardiac arrhythmia

*In Chao it is not reported how outcome was defined or measured but given the authors were using the same database as Suenari, we have assumed outcomes were measured in the same way.

†In Kim, Li and Hu W-S the percentage of patients related to sex, diabetes, hypertension, heart failure was calculated from reported values categorised by incident AF or not.

AF, atrial fibrillation; AFl, atrial flutter; BMI, body mass index; BP, blood pressure; ClalitHS, Clalit Health Services; CN, China; CPRD, Clinical Practice Research Datalink; D, derivation; EHR, electronic health records; EV, external validation; F/U, follow-up; ICD, International Classification of Diseases; ICPC-1, International classification of Primary care version 1 diagnostic codes; IL, Israel; KR, Republic of Korea; MHS, Maccabi Healthcare Services; N/A, not available; NHIRD, National Health Insurance Research Database; NHIS-HEALS, National Health Insurance Service - Health screening Cohort; NHIS-NSC, National Health Insurance Service-based National Sample Cohort; Nivel-PCD, Netherlands Institute for Health Services Research Primary Care Database; NL, Netherlands; TW, Taiwan; YMID, Yunnan Medical Insurance Database.

**Table 2:** Characteristics of included prediction models

Model	Study	Predicted outcome	Number of predictors	Derivation EHR cohort (country)	External validation EHR cohort (country)
**Models originally derived for another purpose but tested for prediction of incident AF**					
CHADS_2_	Gage 2001^37^	Stroke risk	5	–	ClalitHS (IL) NHIRD (TW) NHIS-HEALS (KR) NHIS-NSC (KR) YMID (CN)
CHA_2_DS_2_-VASc	Lip *et al* 38	Stroke risk	7	–	ClalitHS (IL) Nivel-PCD (NL) NHIS-HEALS (KR) NHIS-NSC (KR) YMID (CN)
HATCH	de Vos *et al* 36	Progression to persistent AF	5	–	NHIRD (TW) NHIS-HEALS (KR) NHIS-NSC (KR) YMID (CN)
**Regression model derived in a prospective cohort design**					
CHARGE-AF	Alonso *et al* 48	Incident AF or AFl	11	–	CPRD (UK) Nivel-PCD (NL)
**Regression models derived in EHR**					
C_2_HEST	Li *et al* 31	Incident AF	6	YMID (CN)	NHIRD (TW) NHIS-HEALS (KR)
MHS	Aronson *et al* 26	Incident AF or AFl	10	MHS (IL)	N/A
**Machine learning models derived in EHR**					
CPRD	Hill *et al* 28	Incident AF or AFl	100	CPRD (UK)	Discover (UK)
NHIRD	Hu W-S *et al* 29	Incident AF	19	NHIRD (TW)	N/A
NHIS-NSC	Kim *et al* 35	Incident AF or AFl	22	NHIS-NSC (KR)	N/A

AF, atrial fibrillation; AFl, atrial flutter; CHADS_2_, Congestive heart failure, Hypertension, Age>75, Diabetes mellitus, prior Stroke or transient ischaemic attack [two points]; CHA_2_DS_2_-VASc, Congestive heart failure, Hypertension, Age>75 [2 points], Stroke/transient ischaemic attack/thromboembolism [two points], Vascular disease, Age 65–74, Sex Category; CHARGE-AF, Cohorts for Heart and Ageing Research in Genomic Epidemiology; C_2_HEST, Coronary artery disease/Chronic obstructive pulmonary disease [one point each], Hypertension, Elderly (Age≥75, 2 points), Systolic heart failure, Thyroid disease (hyperthyroidism); ClalitHS, Clalit Health Services; CN, China; CPRD, Clinical Practice Research Datalink; EHR, electronic health records; HATCH, Hypertension, Age, stroke or Transient ischemic attack, Chronic obstructive pulmonary disease, Heart failure; IL, Israel; KR, Republic of Korea; MHS, Maccabi Healthcare Services; N/A, not available; NHIRD, National Health Insurance Research Database; NHIS-HEALS, National Health Insurance Service - Health screening Cohort; NHIS-NSC, National Health Insurance Service-based National Sample Cohort; Nivel-PCD, Netherlands Institute for Health Services Research Primary Care Database; NL, Netherlands; TW, Taiwan; UK, United Kingdom; YMID, Yunnan Medical Insurance Database.

The total number of participants in the included studies was 17 889 536. Cohort size ranged from 96 778 to 2 994 837. The mean age varied from 41.3 years to 65.7 years and the proportion of female participants ranged from 47.3% to 54.7%. The mean follow-up ranged from 2.9 years to 10.9 years. The incidence of AF during follow-up ranged from 0.2% to 5.8%.

### Characteristics of included prediction models

The included studies reported data on nine multivariable prediction models (table 2). Three models had originally been derived for a purpose other than incident AF prediction.^36–38^ Five models had been derived in community-based EHR; three using machine learning techniques.^28 29 35^ In two of these studies, a range of machine learning techniques had been investigated with the optimum technique chosen by discriminative performance (online supplemental table S2).^28 35^ Among machine learning techniques, random forests were investigated in all three studies^28 29 35^ and neural networks were considered in two.^28 35^ All studies reported a measure of discrimination (either c-statistic or AUROC), but only two studies provided a measure of calibration.^9 26^ Three prediction models— CPRD (Clinical Practice Research Datalink), C_2_HEST (Coronary artery disease/Chronic obstructive pulmonary disease (one point each), Hypertension, Elderly (Age ≥75, two points), Systolic heart failure, Thyroid disease (hyperthyroidism)) and HATCH (Hypertension, Age, stroke or Transient ischemic attack, Chronic obstructive pulmonary disease, Heart failure)—showed a c-statistic greater than 0.75 in an external validation study (online supplemental table S1).^30 33^

**Figure 2:**
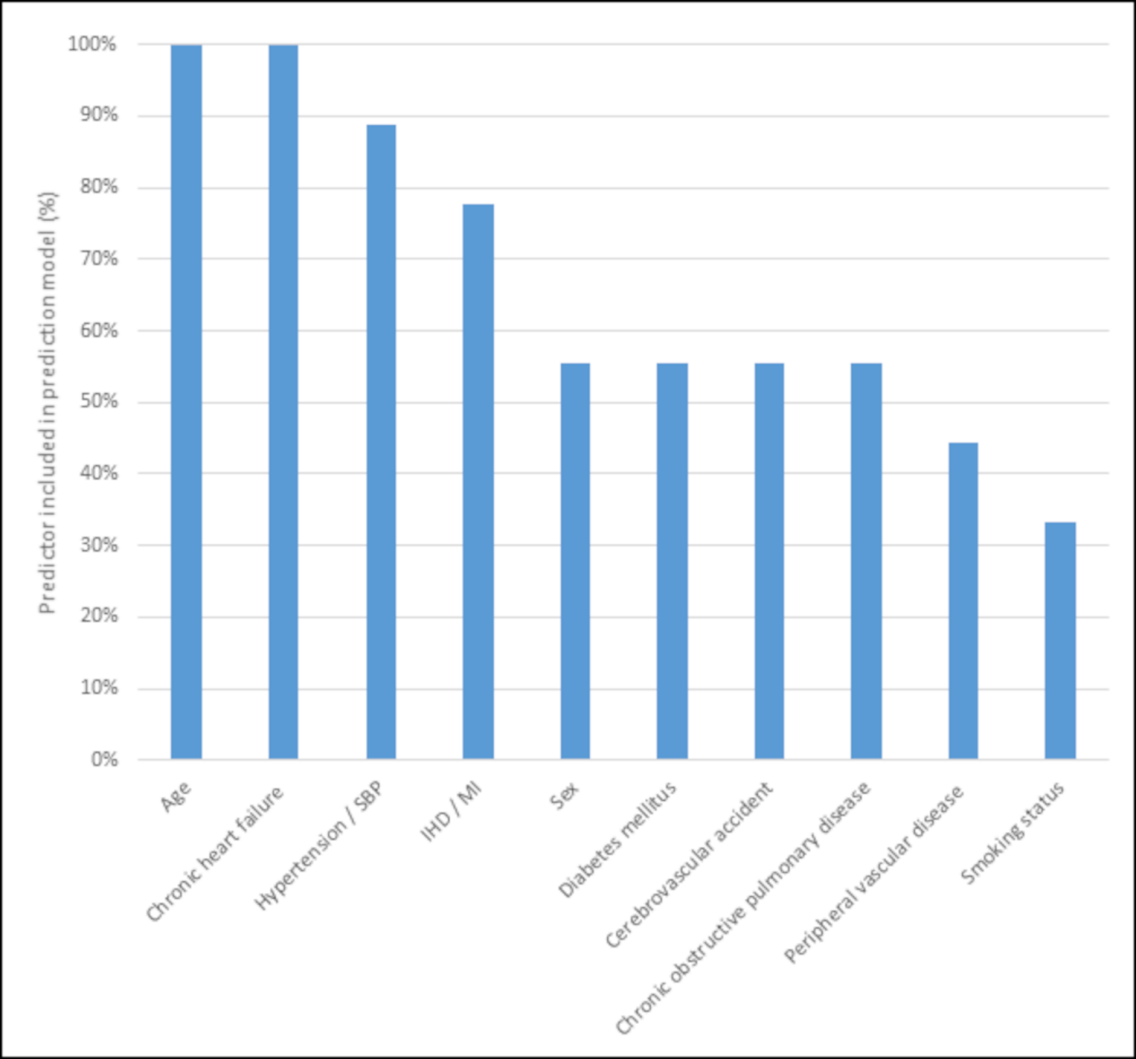
An overview of the ten predictors most frequently incorporated in the prediction models in this study. IHD, ischaemic heart disease; MI, myocardial infarction; SBP, systolic blood pressure.

Online supplemental table 3a, b summarises the variables used. The 10 most frequently included variables are summarised in figure 2. Age and chronic heart failure were the only variables included in every model. The number of variables incorporated into machine learning models was far greater than traditional regression models (table 2). The CPRD model was unique in incorporating time-varying variables (eg, change in body mass index (BMI) between the last two quarters of the year).^28^

Online supplemental figure S1 plots the performance of traditional regression and machine learning models in the development population of each study. Online supplemental table S2 summarises the performance of traditional regression and machine learning techniques during model development in the CPRD and NHIS-NSC data sets. In each case, machine learning produced stronger discriminative performance in the development population.

### Risk of bias assessment

**Figure 3:**
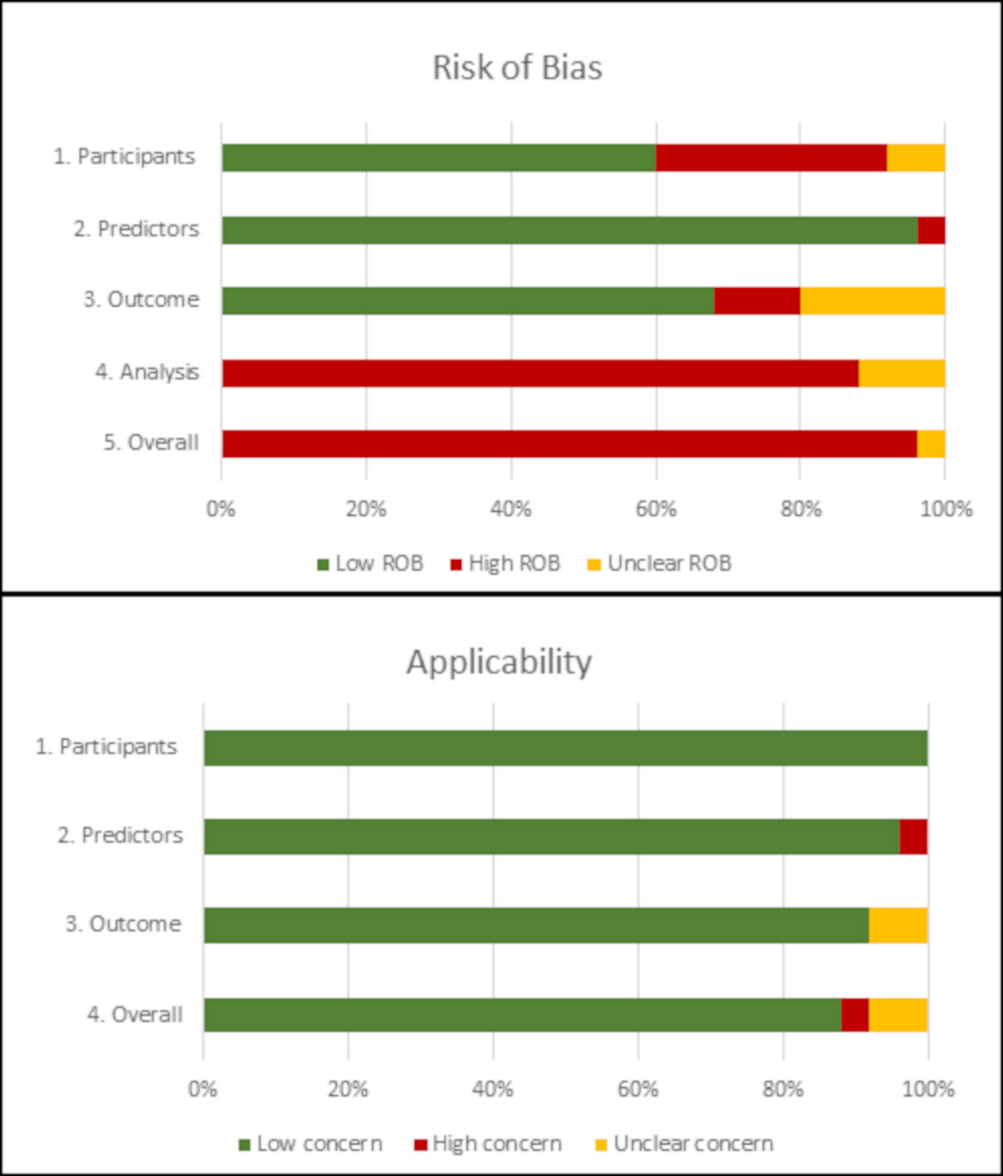
Judgements on the four PROBAST risk of bias domains and three PROBAST applicability domains presented as percentages across all included studies. PROBAST, Prediction model Risk of Bias ASsessment Tool; ROB, risk of bias.

Online supplemental table S4 shows the results of the risk of bias and applicability assessment for each PROBAST domain for each model in the included studies. Figure 3 gives an overall summary of PROBAST domain assessments across all included studies. Overall, 96% of model results were at high risk of bias predominantly driven by high risk of bias in the analysis domain (88%). This resulted from exclusion of participants with missing data from analysis (72%) or not mentioning missing data (16%).

### Meta-analysis

**Figure 4:**
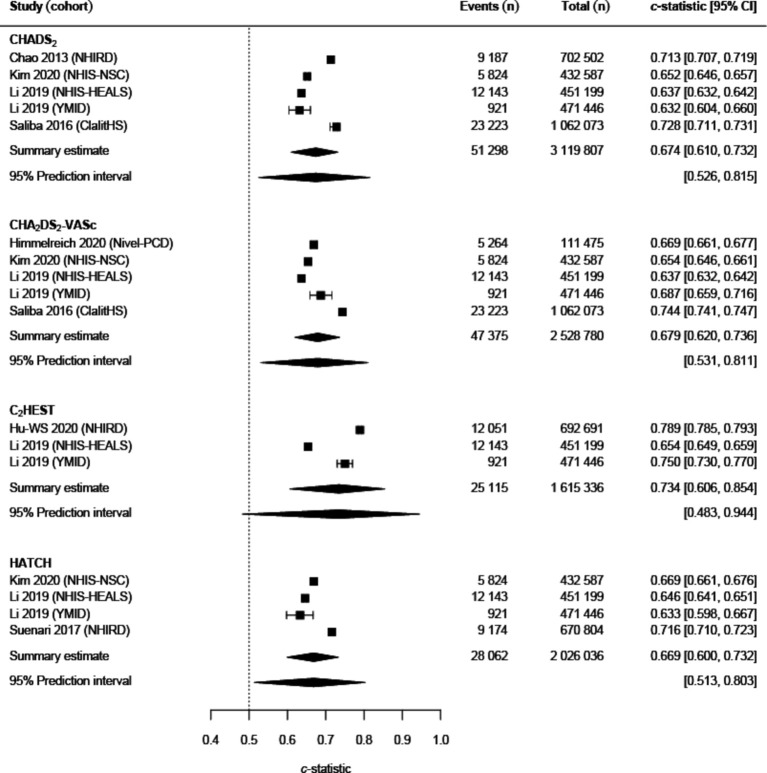
Forest plot of primary analysis of *c*-statistics. C_2_HEST, Coronary artery disease/chronic obstructive pulmonary disease (one point each), Hypertension, Elderly (Age ≥75, 2 points), Systolic heart failure, Thyroid disease (hyperthyroidism); CHADS_2_, Congestive Heart failure, hypertension, Age>75, Diabetes mellitus, prior Stroke or transient ischaemic attack (two points); CHA_2_DS_2_-VASc, Congestive heart failure, Hypertension, Age >75 (2 points), Stroke/transient ischaemic attack/thromboembolism (two points), Vascular disease, Age 65–74, sex category; ClalitHS, Clalit health services; HATCH, Hypertension, Age, stroke or Transient ischemic attack, Chronic obstructive pulmonary disease, and Heart failure; NHIRD, National Health Insurance Research Database; NHIS-HEALS, National Health Insurance Service - Health screening Cohort; NHIS-NSC, National Health Insurance Service-based National Sample Cohort; Nivel-PCD, Netherlands Institute for Health Services Research Primary Care Database; YMID, Yunnan Medical Insurance Database.

Four models were eligible for the primary meta-analysis, incorporating 9 289 959 patients (figure 4). Only C_2_HEST was derived specifically for the purpose of predicting incident AF.^31^ There were three models that resulted in a summary c-statistic with statistically significant 95% PI in our primary meta-analysis: CHADS_2_ (Congestive heart failure, Hypertension, Age >75, Diabetes mellitus, prior Stroke or transient ischemic attack) (summary c-statistic 0.674; 95% CI 0.610 to 0.732; 95% PI 0.526–0.815; n=5 studies; n=3 119 807), CHA_2_DS_2_-VASc (Congestive heart failure, Hypertension, Age >75 (2 points), Stroke/transient ischemic attack/thromboembolism (2 points), Vascular disease, Age 65–74, Sex Category) (summary c-statistic 0.679; 95% CI 0.620 to 0.736; 95% PI 0.531–0.811; n=5 studies; n=2 528 780) and HATCH (summary c-statistic 0.669; 95% CI 0.600 to 0.732; 95% PI 0.513–0.803; n=4 studies; n=2 026 036). There was high heterogeneity, as shown by the wide 95% PIs (figure 4).

Online supplemental table S5 shows the results of the sensitivity analyses. Only CHA_2_DS_2_-VASc maintained a summary c-statistic with statistically significant 95% PI when either restricting the primary analysis to studies with ‘low’ or ‘unclear’ risk of bias for the participants domain of PROBAST, or using later data when a cohort had been analysed multiple times, or excluding data from either of the NHIS-HEALS or NHIS-NSC cohorts. However, when restricting primary analysis to models with ‘low’ or ‘unclear’ risk of bias for overall PROBAST assessment, no models met eligibility for inclusion.

### Certainty of evidence

The initial certainty level of the included prediction modelling studies was set at ‘high’ because the association between the predictors and outcomes was considered irrespective of any causal connection.^39^ The overall certainty level was, however, downgraded to ‘moderate’ and then ‘low’ because of inconsistent results (high heterogeneity) and the large proportion of high overall risk of bias amongst studies. The final overall certainty of ‘low’ implies that our confidence in the effect estimates is limited and further research is very likely to change the effect estimate.

## Discussion

This systematic review and meta-analysis identified nine models that have been derived and/or validated in community-based EHR for incident AF. Five had been derived in EHR for this purpose; three by machine learning methods. Three models (CHADS_2_, CHA_2_DS_2_-VASc and HATCH) produced a summary c-statistic with statistically significant 95% PI for prediction of incident AF despite high heterogeneity. However the summary c-statistics were only 0.669–0.679. For an outcome such as AF that is considered difficult to predict, a *c*-statistic of 0.75 may be adequate for the models to be useful.^40^ This threshold has been achieved by prediction models for incident AF in the community in non-EHR-based external validation studies,^41–43^ as well as in EHR by the machine learning CPRD model.^33^ Furthermore, in sensitivity analyses no model met eligibility for inclusion in meta-analysis if studies at overall high risk of bias were excluded.

A previous meta-analysis investigated prediction models for incident AF that had been derived or validated in community cohorts.^13^ Nevertheless, this review included predominantly carefully curated prospective cohort designs, the results from which will have limited generalisability. In addition, a number of the included models require variables, such as ECG parameters, that are not routinely available in community-based EHR.^44^ The authors found CHA_2_DS_2_-VASc and CHARGE-AF (Cohorts for Heart and Ageing Research in Genomic Epidemiology) resulted in a summary c-statistic with statistically significant 95% PI on meta-analysis. There is conflicting evidence as to how well CHARGE-AF performs in EHR, especially given the incompleteness of structured EHR fields for height, weight and ethnicity,^9 10^ and for our study it did not meet eligibility for inclusion into meta-analysis. Another systematic review summarised a similar selection of prediction models for the detection of AF in the community and externally validated these models head-to-head in a commercial screening cohort.^14^ However, the outcome was prevalence, rather than future incident AF. Both of these reviews predated the emergence of machine learning models in this field, which are summarised for the first time regarding the prediction of incident AF here.

The use of age alone to target screening strategies for incident AF has yet to show a benefit for systematic versus opportunistic screening, which is reflected in international guidelines.^2 7^ Prediction models could target screening and if implemented through primary care EHR would minimise extra resources. The use of CHA_2_DS_2_-VASc for prediction of incident AF has advantages given it uses variables available with high completeness in primary care EHR and would simultaneously provide an assessment of stroke risk as an indicator of eligibility for anticoagulation. Even so, there are a number of limitations. First, the discriminative performance was only moderate, overall certainty in the estimate effects was ‘low’ and the vast majority of studies were at high risk of bias. Second, it has predominantly been validated in Asia Pacific countries, where cohorts had different baseline characteristics compared with European counterparts. Third, it was outperformed by CHARGE-AF and C_2_HEST when compared head-to-head in individual external validation studies.^9 31^

Efforts may be best served to develop and externally validate novel prediction models for incident AF in community-based EHR. These data sources offer large samples sizes, providing the opportunity to investigate a larger number of predictors and use novel techniques. Machine learning models in this review showed strong discriminative performance in development data sets but were not included in meta-analysis due to a sparsity of external validation.

This study has a number of strengths. We had a comprehensive search strategy and thorough analysis approach. We included any model that had been used to predict the risk of incident AF, which allowed us to include models that were not originally intended for predicting AF but may have merits. We only included models that had been tested in databases relevant to the general population, which ensures the applicability of our results for screening in a primary care setting. We also did not present meta-regression or subgroup meta-analysis to investigate heterogeneity between studies based on study-level characteristics or subgroups in the absence of available individual patient data given that such analyses would be prone to ecological bias.^45^

There are limitations to our study. Meta-analysis of model calibration performance was prohibited by poor reporting. We did not assess for ‘reporting biases’ visually through a funnel plot for several reasons. First, some studies reported multiple models in the same cohort so incorporating all these data points would skew the plot; second, producing funnel plots for individual models would not be informative as there would be too few data points; third the sample sizes for all included studies was very large making small-study effects less likely. The vast majority of studies was at high risk of bias, which is consistent with previous literature on clinical prediction models due to limitations in conduct and reporting.^46^ We restricted our search to studies written in English, though this has not been found to lead to significant bias.^47^ Finally, routinely collected databases are associated with a number of potential biases relating to their retrospective, observational nature.

## Conclusions

In this systematic review with meta-analysis, we identified nine multivariable prediction models relevant to screening for incident AF using community-based EHR. On meta-analysis three models produced a summary c-statistic with statistically significant 95% PI, but discriminative performance was only moderate. At present, due to a combination of high risk of bias and inconsistency, there is no high-performing prediction model for incident AF using primary care EHR. Future research could aim to develop models in primary care EHR using machine learning, but must better handle missing data, report calibration and provide external validation.

**Key messages**

**What is already known on this subject?**

- Without a means of stratifying high-risk patients, opportunistic screening is more cost-effective than systematic screening. Several models have been derived for predicting incident atrial fibrillation in the community; predominantly through structured follow-up of prospective cohorts. Community-based electronic health records offer a potential route for far-reaching and cost-effective implementation of such models, but the utility of prediction models in this resource is unknown.

**What might this study add?**

- In this systematic review and meta-analysis we found that models initially derived for other purposes have been tested most frequently for predicting incident atrial fibrillation in community-based electronic health records, but only show moderate and variable performance. There is high heterogeneity between studies and most failed to adequately handle missing data or report calibration. Models derived using machine learning in community-based electronic health records shows promising performance during model development.

**How might this impact on clinical practice?**

- This study suggests that none of the available prediction models are, at present, suitable for targeting screening for atrial fibrillation in the community using electronic health records. Models derived by machine learning could provide improved performance, but require external validation and clinical impact assessment.

## Data Availability

Data are available upon reasonable request. Technical appendix, statistical code and dataset are available from the corresponding author at r.nadarajah@leeds.ac.uk.
