# Supplementary material for: Prediction of incident atrial fibrillation in community-based electronic health records: a systematic review with meta-analysis
Source: Heart. 2021 Oct 4;108(13):1020–9. doi: 10.1136/heartjnl-2021-320036 (PMC9209680; doi:10.1136/heartjnl-2021-320036)
Supplement: Supplementary data [file heartjnl-2021-320036supp001.pdf]

Supplementary material to Nadarajah R, Alsaeed E, Hurdus B et al. "Prediction of incident atrial fibrillation using community-based electronic health records: a systematic review with meta-analysis"

SUPPLEMENTARY METHODS

Formulation of research question using CHARMS (CHecklist for critical Appraisal and data extraction for systematic Reviews of prediction Modelling Studies):

CHARMS key items to guide framing of review, search strategy and study inclusion and exclusion criteria	Comments for this systematic review
Prognostic versus diagnostic prediction model	Prognostic prediction model
Intended scope of the review	Models to inform referral for diagnostic testing
Types of Prediction modelling studies	Prediction model development without external validation in independent data, prediction model development with external validation in independent data, external model validation, possibly with model updating
Target population to whom the prediction model applies	Adults in the general population who have a primary care or community electronic health record
Outcome to be predicted	Specific future event, diagnosis of atrial fibrillation
Time span of prediction	Any time interval
Intended moment of using the model	Models to be used in adults in primary care using electronic health records to predict risk of development of atrial fibrillation in the future, and inform targeted screening

Nadarajah R, *et al. Heart* 2022; 108:1020–1029. doi: 10.1136/heartjnl-2021-320036

Search Terms and search strategy with full results

This search was adapted from *Poorthuis et al* and *Ammemwerth et al*.[1 2]

Database(s): **Ovid MEDLINE(R) ALL** 1946 to March 23, 2021
Search Strategy:

#	Searches	Results
1	atrial fibrillation/ or atrial flutter/	61138
2	(atrial fibrillation or atrial flutter).ti,ab.	77059
3	1 or 2	90196
4	ROC Curve/ or (stratification or discrimination or discriminate or c-statistic or c statistic or Area under the curve or Calibration or Indices or Algorithm or Multivariable).ti,ab.	852681
5	Mass screening/ or Screen*.ti,ab.	823683
6	Prevalence/ or prevalenc*.ti,ab. or incidence/ or incidenc*.ti,ab.	1568534
7	population/ or population*.ti,ab.	1832889
8	5 or 6 or 7	3645965
9	(communit* or data*).ti,ab.	4711739
10	(general adj3 population).ti,ab.	122216
11	database/ or dataset/	1216
12	(Electronic Health Record* or electronic medical record* or electronic personal record* or electronic patient record* or personal health record* or personal medical record* or computer health record* or computer medical record* or computer patient record* or ehr? or phr? or ephr? or emr? or paehr?).ti,ab.	47628
13	Electronic Health Records/ or exp medical records systems computerized/ or exp health records personal/	43724
14	Primary Health Care/ or (primary care or general practic*).ti,ab.	184646
15	9 or 10 or 11 or 12 or 13 or 14	4935714
16	3 and 4 and 8 and 15	1342
17	limit 16 to (english language and humans)	1072

Nadarajah R, *et al. Heart* 2022; 108:1020–1029. doi: 10.1136/heartjnl-2021-320036

Database(s): **Embase Classic+Embase** 1947 to 2021 March 23
Search Strategy:

#	Searches	Results
1	exp heart atrium fibrillation/ or exp atrial fibrillation/ or exp heart atrium flutter/	93727
2	(atrial fibrillation or atrial flutter).ti,ab,kw.	143413
3	1 or 2	166612
4	predict.ti.	78253
5	(validat* or rule*).ti,ab.	1059357
6	(predict* and (outcome* or risk* or model*)).ti,ab.	1313986
7	((history or variable* or criteria or scor* or characteristic* or finding* or factor*) and (predict* or model* or decision* or identif* or prognos*)).ti,ab.	4704335
8	decision*.ti,ab. and statistical model/	7315
9	(decision* and (model* or clinical*)).ti,ab.	276542
10	(prognostic and (history or variable* or criteria or scor* or characteristic* or finding* or factor* or model*)).ti,ab.	370949
11	(stratification or discrimination or discriminate or c statistic or "area under the curve" or auc or calibration or indices or algorithm or multivariable).ti,ab.	1183028
12	receiver operating characteristic/	141783
13	4 or 5 or 6 or 7 or 8 or 9 or 10 or 11 or 12	6613249
14	exp mass screening/	262462
15	Screening.ab,ti,kw.	811498
16	exp prevalence/	814190
17	Prevalence.ab,ti,kw.	959326
18	exp incidence/	533091
19	Incidence.ab,ti,kw.	1207644
20	14 or 15 or 16 or 17 or 18 or 19	3136647
21	(communit* or data*).ti,ab.	6555772
22	(general adj3 population).ti,ab.	184176
23	database/ or dataset/	450631
24	Electronic Health Records/ or (electronic health record* or electronic medical record* or electronic personal record* or electronic patient record* or personal health record* or personal medical record* or computer health	94165

Nadarajah R, *et al. Heart* 2022; 108:1020–1029. doi: 10.1136/heartjnl-2021-320036

	record* or computer medical record* or computer patient record*).ti,ab. or (ehr? or phr? or ephr? or emr? or paehr?).ti,ab.	
25	21 or 22 or 23 or 24	6816789
26	3 and 13 and 20 and 25	7804
27	letter.pt. or letter/	1179167
28	note.pt.	848283
29	conference abstract.pt.	4066914
30	editorial.pt.	690770
31	case report/ or case study/	2780774
32	(letter or comment*).ti.	217544
33	27 or 28 or 29 or 30 or 31 or 32	9080295
34	animal/ not human/	1523407
35	nonhuman/	6523962
36	exp animal experiment/	2699975
37	exp experimental animal/	749370
38	animal model/	1434020
39	exp rodent/	4130266
40	(rat or rats or mouse or mice).ti.	1709912
41	34 or 35 or 36 or 37 or 38 or 39 or 40	9318330
42	33 or 41	17366453
43	26 not 42	3796
44	limit 43 to english language	3636

Inclusion and exclusion criteria pertaining to variables incorporated in models

We have reviewed the text referred to by the reviewer and agree that we have not defined inclusion and exclusion criteria with regards to the variables incorporated in models. Given issues of space we have added these in supplementary material, which is copied below (without references), and referenced them in the main manuscript.

In this review we were interested in models that could use structured 'coded' data in community-based electronic health records or administrative claims databases. To make screening in the community for AF more cost-effective and feasible the model would use variables that are available, calculate the risk automatically, and require minimal additional visits for baseline risk stratification. We only considered the use of structured 'coded' data as the technology for natural language processing to extract free text into 'coded' data is too immature for widespread clinical use. We used examples of primary care or population-based health information databases across the world to define the variables most likely to be coded or extractable, accepting that there will be some variation.

The information that was considered likely to be available in community-based data sources ± linkages (depending on whether the original purpose of the database was documentation of clinical care, epidemiological surveillance, or health system planning) were:

- Sociodemographic variables including but not limited to age, sex, ethnicity and indices of multiple deprivation.
- Disease conditions and procedures including but not limited to other cardiovascular diseases, diabetes mellitus, chronic lung disease, renal disease, inflammatory disease, cancer, hypothyroidism and surgical procedures.
- Clinical assessments including but not limited to heart rate, systolic and diastolic blood pressure, height, weight and body mass index.
- Medications prescribed including but not limited to antihypertensives, statins, antidepressants, anxiolytics/hypnotics and antipsychotics.
- Lifestyle factors including but not limited to smoking status and alcohol consumption.
- Simple laboratory tests and biomarkers including but not limited to total, high-density lipoprotein and low-density lipoprotein cholesterol, triglycerides, creatinine, c-reactive protein, erythrocyte sedimentation rate.
- Referrals

We excluded the following types of variables that are either not routinely available as structured codes, or are very rarely tested for in clinical practice and so are not generalizable:

- Analysis of electrocardiograph (ECG) parameters (e.g. PR interval, QRS duration, p-wave duration).
- Analysis of advanced diagnostic testing such as echocardiography parameters (e.g. left atrial dimensions, left ventricular end-diastolic diameter).
- Genetic markers and specialised (laboratory) tests (e.g. midregional sequence of pro-atrial natriuretic peptide).

Nadarajah R, *et al. Heart* 2022; 108:1020–1029. doi: 10.1136/heartjnl-2021-320036

PROBAST: Justifications for assessments for specific signalling questions

Risk of bias

Domain 1: Participants
Signalling question 2: Were all inclusions and exclusions of participants appropriate?

We assessed risk of bias based on the comorbidities or age cut-offs used for exclusion. With regards to comorbidities when studies excluded patients with valvular heart disease or valve surgery from their population the signalling question was marked as 'N' and the overall domain as 'high' risk of bias. We believe valvular heart disease is a common cardiovascular condition and a strong risk factor for atrial fibrillation.[3]

With regards to age the appropriateness of cut offs was decided by the expected prevalence of atrial fibrillation in the general based on available literature. The highest prevalence is between 65-85 years.[4] In American cohorts of European ancestry, the prevalence has been found to be 0.2% between 40-50 years.[4] In UK population databases the prevalence between 40-49 years has been found to be 0.7%.[4] In Korea and Japan the prevalence between 40-49 years has been found to be 0.2%.[4] In an Israeli population, from Clalit Health Services (CHS), the prevalence was approximately 0.6% below 50 years compared to 15.2% in age over 85 years.[5] Thus overall the consensus was that excluding patients below the age of 50 years would not exclude a subgroup that would alter the performance of the prediction model for the intended target population. In terms of an exclusion criteria at the top of the age stratum it was considered that excluding anyone below 95 years would be inappropriate based on a number of reasons. First, prevalence is very high over 85 years;[5] second, age has been frequently identified as a risk predictor for both atrial fibrillation and stroke secondary to atrial fibrillation;[6] third, 95 years was an upper limit on age for community prospective cohort designs that have been were used to develop prediction models for incident AF,[7 8] and finally there is significant potential in age greater than 85 years for benefit from anticoagulation.[6 9] Thus if a study included the age range 50 – 95 years this contributed to the signalling question being marked as 'Y'.

Domain 4: Analysis
Signalling question 4: Were participants with missing data handled appropriately?

We assessed risk of bias for missing data by assuming that if missing data was not mentioned at all that it was likely missing but not taken account for and thus marked as 'N or PN' and thus 'high' risk of bias. If there was any extent of missing data and methods had not been used to impute or an analysis had not been made to assess whether inclusion of missing values would have made a difference to performance measures, then the signalling question was marked as 'N or PN' and the overall domain as 'high' risk of bias.

Applicability

Domain 1: Participants
Even if the criteria for participant exclusion led to a high risk of bias, for example from exclusion of mitral valve stenosis or prosthetic heart valves, the cohort could still be applicable to the research question if this was a small proportion of the overall population. In such a case if the sample size was very large and derived from on population or primary care databases these were judged to be low concern for applicability for our target population.

Nadarajah R, *et al. Heart* 2022; 108:1020–1029. doi: 10.1136/heartjnl-2021-320036

Studies excluded that met a number of inclusion criteria but were based in electronic health records housed in a secondary care institution, pertaining to a secondary care population or that include variables or investigations measured in secondary care.

1. Brunner, K. J., et al. (2014). Clinical predictors of risk for atrial fibrillation: implications for diagnosis and monitoring. Mayo Clinic Proceedings, Elsevier.
2. Chua, W., et al. (2019). "Data-driven discovery and validation of circulating blood-based biomarkers associated with prevalent atrial fibrillation." European Heart Journal 40(16): 1268-1276.
3. Hulme, O. L., et al. (2019). "Development and validation of a prediction model for atrial fibrillation using electronic health records." JACC Clinical Electrophysiology 5(11): 1331-1341.
4. Khurshid, S., et al. (2020). "Performance of Atrial Fibrillation Risk Prediction Models in Over Four Million Individuals." Circulation: Arrhythmia 14: e008997.
5. Kolek, M. J., et al. (2016). "Evaluation of a prediction model for the development of atrial fibrillation in a repository of electronic medical records." JAMA Cardiol. 1(9): 1007-1013.
6. Tiwari, P., et al. (2020). "Assessment of a machine learning model applied to harmonized electronic health record data for the prediction of incident atrial fibrillation." JAMA network open 3(1): e1919396-e1919396.
7. Volgman, A. S., et al. (2019). "Risk factors for symptomatic atrial fibrillation-analysis of an outpatient database." Journal of Atrial Fibrillation 12(1).
8. Tischer, T. S., et al. (2015). "Prevalence of atrial fibrillation and the HATCH score." Herz 40(5): 803-808.

Studies excluded that met a number of inclusion criteria but diagnosis of atrial fibrillation was based solely on screening follow up examination without using community or hospital diagnoses; so not comparable to routinely-collected electronic health records

1. Hamada, R. and S. Muto (2019). "Simple risk model and score for predicting of incident atrial fibrillation in Japanese." J Cardiol 73(1): 65-72.
2. Ding, L., et al. (2017). "Incidence of atrial fibrillation and its risk prediction model based on a prospective urban Han Chinese cohort." Journal of Human Hypertension 31(9): 574-579.

Study identified through backward citation that met a number of inclusion criteria searching but participant inclusion was restricted to participants exactly at 65 years, 70 years and 75 years and thus excluded such a range of ages (64 years and below, 66-74 years, 76 years and above) that it was unrepresentative of a general primary care population

1. Lip, G. Y., et al. (2020). "Evaluation of the C2HEST risk score as a possible opportunistic screening tool for incident atrial fibrillation in a healthy population (from a nationwide Danish cohort study)." The American Journal of Cardiology 125(1): 48-54.

Bayesian meta-analysis of *c*-statistic / AUROC

All Bayesian meta-analysis models assume random effects by default. Results are based on the posterior median. Prediction intervals are directly obtained from the corresponding posterior quartiles. The standard model for random effects meta-analysis assumes that the 'true' performance is normally distributed within and across studies.[10] Within-study normality of performance estimates can be justified with this selection of included studies because they are all large. *Snell et al.* showd that the between-study distribution of the *c*-statistic on the original scale is not normally distributed when there is variability in the predictor effect across studies (which is likely in this selection of studies as they include different populations, and adopt slightly different definitions for predictors).[10] They found that the logit scale is more appropriate for the estimation of prediction interval. Consequently we used the "valmeta" function of the "metamisc" package in R software which applies a logit transformation to the *c*-statistic prior to calculation of summary *c*-statistic and prediction interval.[11]

For appropriate prior distributions we borrowed from earlier work by *Debray et al.* which recommended a half Student-*t* distribution with location *m*, scale σ, and *v* degrees of freedom where we set $m = 0$ and define σ equal to the largest empirical value of $\hat{\tau}$ (to allow for more extreme values of heterogeneity).[12] These hyperparameter values allow to penalise the extent of between-study heterogeneity when the number of included validation studies is low.[12] Further we also used $v = 3$ to ensure that the variance $\sigma^2\, v/(v\text{-}2)$ exists and samples of τ were truncated above 10 to rule out unreasonable values. Thus the resulting priors are given as τdiscr ~ Student−$t(0, 0.5^2, 3)$T[0.10] which has been shown to allow for large but realistic values for between-study heterogeneity.[12]

Nadarajah R, *et al. Heart* 2022; 108:1020–1029. doi: 10.1136/heartjnl-2021-320036

GRADE: Standards used for judging criteria for downgrading and upgrading certainty of evidence

The certainty of the evidence was graded as 'high' (further research is very unlikely to change our confidence in the effect estimate), 'moderate' (further research is likely to have an important impact on our confidence in the effect estimate), 'low' (further research is very likely to have an important impact on our confidence in the effect estimate and is likely to change the estimate) or 'very low' (any estimate of effect is very uncertain).

The initial certainty level of the included prediction modelling studies was set at 'high' because the association between the predictors and outcomes was considered irrespective of any causal connection.[13] Eight criteria were considered to further downgrade or upgrade the certainty of the evidence; five criteria which might downgrade the overall certainty of the evidence (methodological limitations of the study, indirectness, imprecision, inconsistency and likelihood of publication bias) and three which might potentially upgrade the overall certainty of the evidence (large effect, dose-response relation in the effect, and opposing plausible residual bias or confounding).

Methodological limitations of the studies were assessed by considering the overall risk of bias judgement across studies based on the overall PROBAST risk of bias assessment. Indirectness was assessed by making a global judgement on how dissimilar the research evidence is to the research question at hand (in terms of population and outcomes across studies).

Indirectness was assessed through concerns regarding the applicability of each included study from PROBAST (i.e. when the populations, predictors or outcomes of the study differ from the research question) and an overall judgement across studies was made.

Imprecision was assessed by considering the optimal total number of events across all studies. A minimum threshold of 10 events per variable was considered as the minimum required in regression modelling development studies, and 100 when machine learning methods had been used.[14 15] For external validation studies a minimum sample size of at least 200 events was less concerning for imprecision.[16] Results may also be imprecise when the 95% confidence intervals of c-statistic of all studies or of the largest studies include insufficient discrimination performance (0.5).

A global judgement on inconsistency was evaluated through the consistency of the model discrimination performance and the range of the 95% PI as a statistical measure of heterogeneity. Widely differing estimates of the c-statistic indicated inconsistency or if the 95% PI of the summary c-statistic was wide and included 0.5.

Publication bias was suspected when the body of evidence consisted of only positive studies from small sample sizes or all studies were funded by industry.

A large magnitude of effect (i.e. highly discriminatory predictive performance) was considered if the c-statistic exceeded 0.7 in the majority of studies.[17] Since this review was not focused on drugs or pharmaceutical agents, assessing a dose-response gradient was not applicable here. Finally, we only included studies that described a multivariable prediction model and thus making a judgement whether all plausible confounders and biases were accounted for and may lead to an underestimated association is not applicable here

Nadarajah R, *et al. Heart* 2022; 108:1020–1029. doi: 10.1136/heartjnl-2021-320036

SUPPLEMENTARY RESULTS

Supplementary Table S1: Outcomes of studies reporting on prediction models

Prediction Model	Study aim	Study (cohort)	Observed AF/total population (%)	Discrimination		Calibration		Follow up duration (years)	RoB Partici-pants domain	RoB Overall
				c-statistic	95%CI	p-value of GOF test	O:E ratio			
**Models originally derived for another purpose but tested for prediction of incident atrial fibrillation**										
$CHADS_2$	EV	Chao 2013 (NHIRD)	9,187 / 702,502 (1.30)	0.713	0.707 - 0.719	N/A	N/A	10	L	H
	EV	Saliba 2016 (ClalitHS)	23,223 / 1,062,073 (2.19)	0.728	0.711 - 0.731†	N/A	N/A	3	U	H
	EV	Li 2019 (YMID)	921 / 471,446 (0.20)	0.632	0.604 - 0.660	N/A	N/A	11	L	H
	EV	Li 2019 (NHIS-HEALS)	12,143 / 451,199 (2.69)	0.637	0.632 - 0.642	N/A	N/A	11	H	H
	EV	Kim 2020 (NHIS-NSC)	5,824 / 432,587 (1.35)	0.652	0.646 - 0.657	N/A	N/A	5	H	H
CHA_2DS_2-VASc	EV	Saliba 2016 (ClalitHS)	23,223 / 1,062,073 (2.19)	0.744	0.741 - 0.747	N/A	N/A	3	U	H
	EV	Li 2019 (YMID)	921 / 471,446 (0.20)	0.687	0.659 - 0.716	N/A	N/A	11	L	H
	EV	Li 2019 (NHIS-HEALS)	12,143 / 451,199 (2.69)	0.637	0.632 - 0.642	N/A	N/A	11	H	H
	EV	Himmelreich 2020 (Nivel-PCD)	5,264 / 111,475 (4.72)	0.669	0.661 - 0.677	N/A	N/A	5	L	H
	EV	Kim 2020 (NHIS-NSC)	5,824 / 432,587 (1.35)	0.654	0.646 - 0.661	N/A	N/A	5	H	H
HATCH	EV	Suenari 2017 (NHIRD)	9,174 / 670,804 (1.40)	0.716	0.710 - 0.723	N/A	N/A	9	L	U
	EV	Li 2019 (YMID)	921 / 471,446 (0.20)	0.633	0.598 - 0.667	N/A	N/A	11	L	H
	EV	Li 2019 (NHIS-HEALS)	12,143 / 451,199 (2.69)	0.646	0.641 - 0.651	N/A	N/A	11	H	H
	EV	Kim 2020 (NHIS-NSC)	5,824 / 432,587 (1.35)	0.669	0.661 - 0.676	N/A	N/A	5	H	H
	EV	Hu-WS 2020 (NHIRD)	12,051 / 692,691 (1.74)	0.771*	0.767 - 0.775	N/A	N/A	14	L	H
**Machine Learning models**										
CPRD	D	Hill 2019 (CPRD)	95,607 / 2,994,837 (3.19)⁺	0.827*	0.826 - 0.828	N/A	N/A	11	L	H

	EV	Sekelj 2020 (Discover)	17,880 / 604,135 (2.96)	0.870*	0.867 - 0.873	N/A	N/A	8	L	H
NHIRD	D[#]	Hu-WS 2019 (NHIRD)	14,212 / 682,237 (2.08)	0.948	0.947 - 0.949	N/A	N/A	14	L	H
NHIS-NSC[§]	D	Kim 2020 (NHIS-NSC)	5,824 / 432,587 (1.35)	0.845	0.837 - 0.853	N/A	N/A	5	H	H
**Regression Models derived in electronic health records**										
C_2HEST	D	Li 2019 (YMID)	921 / 471,446 (0.20)	0.750	0.730 - 0.770	N/A	N/A	11	L	H
	EV	Li 2019 (NHIS-HEALS)	12,143 / 451,199 (2.69)	0.654	0.649 - 0.659	N/A	N/A	11	H	H
	EV	Hu-WS 2020 (NHIRD)	12,051 / 692,691 (1.74)	0.790*	0.785 - 0.793	N/A	N/A	14	L	H
MHS	D	Aronson 2018 (MHS)	5,660 / 96,778 (5.80)	0.743	0.737 - 0.749	N/A	0.970**	10	L	H
**Regression model derived in a prospective cohort design**										
CHARGE-AF	EV	Hill 2019 (CPRD)	95,607 / 2,994,837 (3.19)[+]	0.725*	0.723 - 0.727	N/A	N/A	11	L	H
	EV	Himmelreich 2020 (Nivel-PCD)	5,264 / 111,475 (4.72)	0.736	0.727 - 0.744	0.001	0.69	5	L	H

AF, Atrial Fibrillation; CHADS$_2$, Congestive heart failure, Hypertension, Age >75, Diabetes mellitus, prior Stroke or transient ischemic attack [2 points]; CHA$_2$DS$_2$-VASc, Congestive heart failure, Hypertension, Age >75 [2 points], Stroke/transient ischemic attack/thromboembolism [2 points], Vascular disease, Age 65-74, Sex Category; CHARGE-AF, Cohorts for Heart and Aging Research in Genomic Epidemiology; C_2HEST, Coronary artery disease / Chronic obstructive pulmonary disease [1 point each], Hypertension, Elderly (Age ≥75, 2 points), Systolic heart failure, Thyroid disease (hyperthyroidism); CI, Confidence Interval; ClalitHS, Clalit Health Services; CPRD, Clinical Practice Research Datalink; D, derivation; EHR, electronic health records; EV, external validation; GOF, goodness-of-fit; H, high; HATCH, Hypertension, Age, stroke or Transient ischemic attack, Chronic obstructive pulmonary disease, Heart failure; L, low; MHS, Maccabi Healthcare Services; NHIRD, National Health Insurance Research Database; NHIS-HEALS, National Health Insurance Service - Health screening Cohort; NHIS-NSC, National Health Insurance Service-based National Sample Cohort; Nivel-PCD, Netherlands Institute for Health Services Research Primary Care Database; N/A, not available; O:E, observed versus expected events; ROB, risk of bias; U, unclear; YMID, Yunnan Medical Insurance Database.

* 95% CI for *c*-statistic not reported in article, so estimated from the reported *c*-statistic according to methods described by Debray *et al.* 2017; ** For Aronson 2018 the reported O:E was extracted by Himmelreich *et al.* 2020; [#] In Hu-WS 2019 the authors do an EV but in a subset of the NHIRD dataset pertaining to secondary care inpatients, preventing us from including this data into this review; † In Saliba 2016 the 95% upper CI for *c*-statistic is reported as 0.725 but this is Ler than the stated *c*-statistic of 0.728, so the 95% upper CI has been estimated from the reported *c*-statistic according to methods described by Debray *et al.* 2017; [+] In Hill 2019 a total of 2,994,837 patients were included in the baseline model with 167,672 included in the time-varying model. The number of events are not differentiated between baseline and time-varying model. This dataset was divided between training (1,996,788) and holdout (998,049) for testing but number of events in each are not reported. For the EV of CHARGE-AF it is not specified which subset of the data is used for validation; [§] In Kim 2020 prediction model development using machine learning was completed both with and without the predictor PM$_{2.5}$ - which is fine particular matter air pollution. In this analysis we have only included the model without PM$_{2.5}$ as it is judged not to be a predictor that would be routinely available in primary care or population EHR.

Nadarajah R, *et al. Heart* 2022; 108:1020–1029. doi: 10.1136/heartjnl-2021-320036

Supplementary Table S2: Performance of machine learning and traditional regression techniques during model development

Technique	Discrimination		Calibration	
	c-statistic	95%CI	p-value of GOF test	O:E ratio
**Hill 2019 (CPRD)**				
Neural network	0.818*	0.817 - 0.819	N/A	N/A
Random forest	0.812*	0.811 - 0.813	N/A	N/A
Support vector machine	0.811*	0.810 - 0.812	N/A	N/A
Logistic LASSO	0.811*	0.810 - 0.812	N/A	N/A
Traditional regression	0.797*	0.796 - 0.798	N/A	N/A
**Hu-WS 2019 (NHIRD)**				
Random forest	0.948	0.947 - 0.949	N/A	N/A
**Kim 2020 (NHIS-NSC)**				
Extreme gradient boosting	0.845	0.837 - 0.853	N/A	N/A
Random forest	0.838	0.830 - 0.846	N/A	N/A
Naïve Bayes	0.833	0.825 - 0.841	N/A	N/A
Deep neural network	0.813	0.800 - 0.826	N/A	N/A
Decision tree	0.801	0.787 - 0.815	N/A	N/A
Support vector machine	0.766	0.757 - 0.775	N/A	N/A
Traditional Regression	0.684	0.675 - 0.693	N/A	N/A

CI, Confidence Interval; CPRD, Clinical Practice Research Datalink; GOF, goodness-of-fit; LASSO, least absolute shrinkage and selection operator; N/A, not available; NHIRD, National Health Insurance Research Database; NHIS-NSC, National Health Insurance Service-based National Sample Cohort; O:E, observed versus expected events.

* 95% CI for *c*-statistic not reported in article, so estimated from the reported *c*-statistic according to methods described by Debray *et al.* 2017

Nadarajah R, *et al. Heart* 2022; 108:1020–1029. doi: 10.1136/heartjnl-2021-320036

Supplementary Table S3a: Baseline variables used in prediction models

Model	Predictors				
	**Patient characteristics**	**Medical History**	**Physical measurements**	**Investigations**	**Other**
**Models originally derived for another purpose but tested for prediction of incident atrial fibrillation**					
$CHADS_2$	Age	Hypertension, CHF, diabetes mellitus, CVA			
CHA_2DS_2-VASc	Age, sex	Hypertension, CHF, stroke/TIA/thromboembolism, vascular disease			
HATCH	Age	Hypertension, CHF, stroke/TIA, COPD			
**Machine Learning models**					
CPRD	Age, sex, race, smoking status	Hypertension, anti-hypertensive medication, CHF, congenital heart disease, MI, LVH, type 1 DM, type 2 DM	Height, weight, BMI, SBP, DBP		
NHIRD	Age (years), age group, sex	Hypertension, CHF, COPD, rheumatological disease, dyslipidaemia, DM, CVA or TIA, sleep disorder, cancer, hyperthyroidism, vascular disease, gout, CKD or ESRD, anaemia			Follow-up duration (years), mean CHA_2DS_2-VASC score
NHIS-NSC*	Age, sex, smoking (pack-year), alcohol	Hypertension, CHF, MI, vascular disease, stroke/TIA, COPD	BMI, SBP	Triglycerides, total cholesterol, HDL cholesterol, LDL cholesterol, eGFR, GGT, fasting blood glucose, Haemoglobin, AST	Socioeconomic status
**Regression Models derived in electronic health records**					
C_2HEST	Age	Hypertension, ischaemic heart disease, CHF, COPD, thyroid disease			
MHS	Age, sex	Anti-hypertensive medication, MI, CHF, peripheral vascular disease, inflammatory disease in a female, COPD	BMI, SBP		
**Regression model derived in a prospective cohort design**					

Nadarajah R, *et al. Heart* 2022; 108:1020–1029. doi: 10.1136/heartjnl-2021-320036

CHARGE-AF	Age, race, smoking status	Anti-hypertensive medication, MI, CHF, DM	Height, weight, SBP, DBP		

Supplementary Table S3b: Time-varying variables in CPRD model of *Hill et al*[18]

Variable	Description of time-varying component
**Patient Characteristics**	
Age	Age in years at start of each 91-day quarter
Sex	Male or female
Race	Known white or other
Smoking status	Known current smoker or other
Height	Latest recorded value
Weight<br>BMI<br>DBP<br>SBP	A new set of predictors was derived using clinical measurements over the year prior to AF date (or equivalent for matched non-AF patients):<br>• latest value recorded in each quarter<br>• difference between latest and earliest values recorded in total<br>• difference between min and max values in each quarter<br>• difference between min and max values across successive quarters<br>• difference between min and max values recorded in total<br>• number of measurements recorded in each quarter<br>• number of measurements recorded in total
**Medical History**	
Hypertension<br>Anti-hypertensive medication<br>CHF<br>Ischaemic heart disease<br>Congenital heart disease<br>MI<br>LVH<br>Type 1 DM<br>Type 2 DM	For each comorbidity, a new set of predictors was derived to indicate whether an event was observed in each quarter over the year prior to AF diagnosis (or equivalent for matched non-AF patients), or at any time prior to this

AF, Atrial Fibrillation; AST, aspartate aminotransferase; BMI, body mass index; $CHADS_2$, Congestive heart failure, Hypertension, Age >75, Diabetes mellitus, prior Stroke or transient ischemic attack [2 points]; CHA_2DS_2-VASc, Congestive heart failure, Hypertension, Age >75 [2 points], Stroke/transient ischemic attack/thromboembolism [2 points]; CHARGE-AF, Cohorts for Heart and Aging Research in Genomic Epidemiology; C_2HEST, Coronary artery disease / Chronic obstructive pulmonary disease [1 point

each], Hypertension, Elderly (Age ≥75, 2 points), Systolic heart failure, Thyroid disease (hyperthyroidism); CHF, chronic heart failure; CKD, chronic kidney disease; COPD, chronic obstructive pulmonary disease; CPRD, Clinical Practice Research Datalink; CVA, cerebrovascular accident; DBP, diastolic blood pressure; DM, diabetes mellitus; eGFR, estimated glomerular filtration rate; ESRD, end-stage renal disease; GGT, gamma glutamyl transferase; HATCH, Hypertension, Age, stroke or Transient ischemic attack, Chronic obstructive pulmonary disease, Heart failure; HDL, high density lipoprotein; L, low; LDL, low density lipoprotein; LVH, left ventricular hypertrophy; MHS, Maccabi Healthcare Services; MI, myocardial infarction; NHIRD, National Health Insurance Research Database; NHIS-HEALS, National Health Insurance Service - Health screening Cohort; NHIS-NSC, National Health Insurance Service-based National Sample Cohort; SBP, systolic blood pressure; TIA, transient ischaemic attack.
* In Kim 2020 prediction model development using machine learning was completed both with and without the predictor $PM_{2.5}$ - which is fine particular matter air pollution. In this analysis we have only included the model without $PM_{2.5}$ as it is judged not to be a predictor that would be routinely available in primary care or population EHR.

Nadarajah R, *et al. Heart* 2022; 108:1020–1029. doi: 10.1136/heartjnl-2021-320036

Supplementary Table S4: Risk of bias and applicability assessment for each PROBAST domain

Study	Model	Aim	RoB Participants	RoB Predictors	RoB Outcome	RoB Analysis	Applicability Participants	Applicability Predictors	Applicability Outcomes	Overall RoB	Overall Applicability
Aronson 2018	MHS	D	L	L	L	H	L	L	L	H	L
Chao 2013	$CHADS_2$	EV	L	L	U	H	L	L	L	H	L
Hill 2019	CPRD	D	L	L	H	U	L	L	L	H	L
Hill 2019	CHARGE-AF	EV	L	L	H	U	L	L	L	H	L
Himmelreich 2020	CHARGE-AF	EV	L	L	L	H	L	L	L	H	L
Himmelreich 2020	CHA_2DS_2-VASc	EV	L	L	L	H	L	L	L	H	L
Hu-WS 2019	NHIRD	D	L	H	L	H	L	H	L	H	H
Hu-WS 2020	C_2HEST	EV	L	L	U	H	L	L	U	H	U
Hu-WS 2020	HATCH	EV	L	L	U	H	L	L	U	H	U
Kim 2020	NHIS-NSC	D	H	L	L	H	L	L	L	H	L
Kim 2020	$CHADS_2$	EV	H	L	L	H	L	L	L	H	L
Kim 2020	CHA_2DS_2-VASc	EV	H	L	L	H	L	L	L	H	L
Kim 2020	HATCH	EV	H	L	L	H	L	L	L	H	L
Li 2019	C_2HEST	D	L	L	L	H	L	L	L	H	L
Li 2019	C_2HEST	EV	H	L	L	H	L	L	L	H	L
Li 2019	$CHADS_2$	EV (YMID)	L	L	L	H	L	L	L	H	L
Li 2019	$CHADS_2$	EV (NHIS-HEALS)	H	L	L	H	L	L	L	H	L
Li 2019	CHA_2DS_2-VASc	EV (YMID)	L	L	L	H	L	L	L	H	L
Li 2019	CHA_2DS_2-VASc	EV (NHIS-HEALS)	H	L	L	H	L	L	L	H	L
Li 2019	HATCH	EV (YMID)	L	L	L	H	L	L	L	H	L
Li 2019	HATCH	EV (NHIS-HEALS)	H	L	L	H	L	L	L	H	L
Saliba 2016	$CHADS_2$	EV	U	L	U	H	L	L	L	H	L
Saliba 2016	CHA_2DS_2-VASc	EV	U	L	U	H	L	L	L	H	L
Sekelj 2020	CPRD	EV	L	L	H	H	L	L	L	H	L
Suenari 2017	HATCH	EV	L	L	L	U	L	L	L	U	L

$CHADS_2$, Congestive heart failure, Hypertension, Age >75, Diabetes mellitus, prior Stroke or transient ischemic attack [2 points]; CHA_2DS_2-VASc, Congestive heart failure, Hypertension, Age >75 [2 points], Stroke/transient ischemic attack/thromboembolism [2 points], Vascular disease, Age 65-74, Sex Category; CHARGE-AF, Cohorts for Heart and Aging Research in Genomic Epidemiology; C_2HEST, Coronary artery disease / Chronic

Nadarajah R, *et al. Heart* 2022; 108:1020–1029. doi: 10.1136/heartjnl-2021-320036

obstructive pulmonary disease [1 point each], Hypertension, Elderly (Age ≥75, 2 points), Systolic heart failure, Thyroid disease (hyperthyroidism); ClalitHS, Clalit Health Service; CPRD, Clinical Practice Research Datalink; D, derivation; EV, external validation; H, high; HATCH, Hypertension, Age, stroke or Transient ischemic attack, Chronic obstructive pulmonary disease, Heart failure; L, low; MHS, Maccabi Healthcare Services; NHIRD, National Health Insurance Research Database; NHIS-HEALS, National Health Insurance Service - Health screening Cohort; NHIS-NSC, National Health Insurance Service (of Korea)-based National Sample Cohort; RoB, risk of bias; U, unclear; YMID, Yunnan Medical Insurance Database.

Nadarajah R, *et al. Heart* 2022; 108:1020–1029. doi: 10.1136/heartjnl-2021-320036

Supplementary Table S5: Sensitivity analyses

Comparison	Summary *c*-statistic	95%CI	95%PI	Studies (n)	Patients (n)
**$CHADS_2$**					
Primary meta-analysis	0.674	0.610-0.732	0.526-0.815	5	3,119,807
Excluding studies with High ROB in participants domain of PROBAST	0.694	0.581-0.798	0.478-0.887	3	2,236,021
Exclude data from NHIS-NSC	0.680	0.595-0.754	0.492-0.836	4	2,687,220
NHIRD data by Hu-WS 2020 not Suenari 2017 and data from NHIS-NSC rather than NHIS-HEALS	0.684	0.606-0.759	0.514-0.843	4	2,668,608
**CHA_2DS_2-VASc**					
Primary meta-analysis	0.679	0.620-0.736	0.531-0.811	5	2,528,780
Excluding studies with High ROB in participants domain of PROBAST	0.702	0.603-0.795	0.510-0.877	3	1,644,994
Exclude data in NHIS-NSC	0.690	0.602-0.758	0.520-0.850	4	2,096,193
NHIRD data by Hu-WS 2020 not Suenari 2017 and data from NHIS-NSC rather than NHIS-HEALS	0.690	0.618-0.760	0.530-0.835	4	2,077,581
**HATCH**					
Primary meta-analysis	0.669	0.600-0.732	0.513-0.803	4	2,026,036
NHIRD data by Hu-WS 2020 not Suenari 2017	0.684	0.586-0.782	0.467-0.880	4	2,047,923
Exclude data from NHIS-NSC	0.668	0.561-0.769	0.460-0.861	3	2,286,140
NHIRD data by Hu-WS 2020 not Suenari 2017 and data from NHIS-NSC rather than NHIS-HEALS	0.696	0.558-0.822	0.436-0.931	3	1,596,724

$CHADS_2$, Congestive heart failure, Hypertension, Age >75, Diabetes mellitus, prior Stroke or transient ischemic attack [2 points]; CHA_2DS_2-VASc, Congestive heart failure, Hypertension, Age >75 [2 points], Stroke/transient ischemic attack/thromboembolism [2 points], Vascular disease, Age 65-74, Sex category; CI, Confidence Interval; HATCH, Hypertension, Age, stroke or Transient ischemic attack, Chronic obstructive pulmonary disease, Heart failure; NHIRD, National Health Insurance Research Database; NHIS-HEALS, National Health Insurance Service - Health screening Cohort; NHIS-NSC, National Health Insurance Service - based National Sample Cohort; PI, Prediction Interval; ROB, Risk of bias.

Nadarajah R, *et al. Heart* 2022; 108:1020–1029. doi: 10.1136/heartjnl-2021-320036

Supplementary Figure S1: Forest plot showing the performance of traditional regression versus machine learning models using the development data from each relevant study

Model	Study	Development method	Events (n)	Total (n)	*c*-statistic [95% CI]
MHS	Aronson 2018	Regression	5 660	96 778	0.743 [0.737, 0.749]
C_2HEST	Li 2019	Regression	921	471 446	0.750 [0.730, 0.770]
CPRD	Hill 2019	Machine learning	95 607	2 994 837	0.827 [0.826, 0.828]
NHIS-NSC	Kim 2020	Machine learning	5 824	432 587	0.845 [0.837, 0.853]
NHIRD	Hu-WS 2019	Machine learning	14 212	682 237	0.948 [0.947, 0.949]

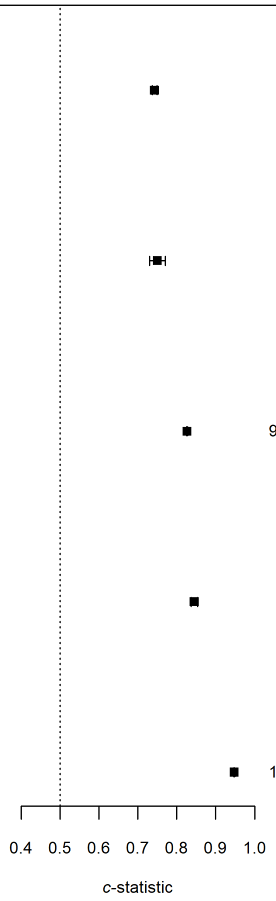

C_2HEST, Coronary artery disease / Chronic obstructive pulmonary disease [1 point each], Hypertension, Elderly (Age ≥75, 2 points), Systolic heart failure, Thyroid disease (hyperthyroidism); CI, Confidence Interval; CPRD, Clinical Practice Research Datalink; D, derivation; MHS, Maccabi Healthcare Services; NHIRD, National Health Insurance Research Database; NHIS-NSC, National Health Insurance Service-based National Sample Cohort.

PRISMA CHECKLIST

Section and Topic	Item #	Checklist item	Location where item is reported
**TITLE**			
Title	1	Identify the report as a systematic review.	1
**ABSTRACT**			
Abstract	2	See the PRISMA 2020 for Abstracts checklist.	2
**INTRODUCTION**			
Rationale	3	Describe the rationale for the review in the context of existing knowledge.	4
Objectives	4	Provide an explicit statement of the objective(s) or question(s) the review addresses.	4
**METHODS**			
Eligibility criteria	5	Specify the inclusion and exclusion criteria for the review and how studies were grouped for the syntheses.	5
Information sources	6	Specify all databases, registers, websites, organisations, reference lists and other sources searched or consulted to identify studies. Specify the date when each source was last searched or consulted.	5
Search strategy	7	Present the full search strategies for all databases, registers and websites, including any filters and limits used.	Supplementary material
Selection process	8	Specify the methods used to decide whether a study met the inclusion criteria of the review, including how many reviewers screened each record and each report retrieved, whether they worked independently, and if applicable, details of automation tools used in the process.	5
Data collection process	9	Specify the methods used to collect data from reports, including how many reviewers collected data from each report, whether they worked independently, any processes for obtaining or confirming data from study investigators, and if applicable, details of automation tools used in the process.	5
Data items	10a	List and define all outcomes for which data were sought. Specify whether all results that were compatible with each outcome domain in each study were sought (e.g. for all measures, time points,	6

Section and Topic	Item #	Checklist item	Location where item is reported
		analyses), and if not, the methods used to decide which results to collect.	
	10b	List and define all other variables for which data were sought (e.g. participant and intervention characteristics, funding sources). Describe any assumptions made about any missing or unclear information.	6
Study risk of bias assessment	11	Specify the methods used to assess risk of bias in the included studies, including details of the tool(s) used, how many reviewers assessed each study and whether they worked independently, and if applicable, details of automation tools used in the process.	6
Effect measures	12	Specify for each outcome the effect measure(s) (e.g. risk ratio, mean difference) used in the synthesis or presentation of results.	6
Synthesis methods	13a	Describe the processes used to decide which studies were eligible for each synthesis (e.g. tabulating the study intervention characteristics and comparing against the planned groups for each synthesis (item #5)).	6
	13b	Describe any methods required to prepare the data for presentation or synthesis, such as handling of missing summary statistics, or data conversions.	6
	13c	Describe any methods used to tabulate or visually display results of individual studies and syntheses.	6
	13d	Describe any methods used to synthesize results and provide a rationale for the choice(s). If meta-analysis was performed, describe the model(s), method(s) to identify the presence and extent of statistical heterogeneity, and software package(s) used.	6
	13e	Describe any methods used to explore possible causes of heterogeneity among study results (e.g. subgroup analysis, meta-regression).	14
	13f	Describe any sensitivity analyses conducted to assess robustness of the synthesized results.	6
Reporting bias assessment	14	Describe any methods used to assess risk of bias due to missing results in a synthesis (arising from reporting biases).	14
Certainty assessment	15	Describe any methods used to assess certainty (or confidence) in the body of evidence for an outcome.	7

Nadarajah R, *et al. Heart* 2022; 108:1020–1029. doi: 10.1136/heartjnl-2021-320036

Section and Topic	Item #	Checklist item	Location where item is reported
**RESULTS**			
Study selection	16a	Describe the results of the search and selection process, from the number of records identified in the search to the number of studies included in the review, ideally using a flow diagram.	8, Figure 1
	16b	Cite studies that might appear to meet the inclusion criteria, but which were excluded, and explain why they were excluded.	Supplementary material
Study characteristics	17	Cite each included study and present its characteristics.	8-12, Table 1 and 2, Figure 2, table S2, S3a, S3b
Risk of bias in studies	18	Present assessments of risk of bias for each included study.	13, Figure 3, table S4
Results of individual studies	19	For all outcomes, present, for each study: (a) summary statistics for each group (where appropriate) and (b) an effect estimate and its precision (e.g. confidence/credible interval), ideally using structured tables or plots.	13, table S1
Results of syntheses	20a	For each synthesis, briefly summarise the characteristics and risk of bias among contributing studies.	13
	20b	Present results of all statistical syntheses conducted. If meta-analysis was done, present for each the summary estimate and its precision (e.g. confidence/credible interval) and measures of statistical heterogeneity. If comparing groups, describe the direction of the effect.	13, figure 4, figure S1
	20c	Present results of all investigations of possible causes of heterogeneity among study results.	N/A
	20d	Present results of all sensitivity analyses conducted to assess the robustness of the synthesized results.	13, table S5
Reporting biases	21	Present assessments of risk of bias due to missing results (arising from reporting biases) for each synthesis assessed.	N/A
Certainty of evidence	22	Present assessments of certainty (or confidence) in the body of evidence for each outcome assessed.	13
**DISCUSSION**			

Nadarajah R, *et al. Heart* 2022; 108:1020–1029. doi: 10.1136/heartjnl-2021-320036

Section and Topic	Item #	Checklist item	Location where item is reported
Discussion	23a	Provide a general interpretation of the results in the context of other evidence.	14-15
	23b	Discuss any limitations of the evidence included in the review.	15
	23c	Discuss any limitations of the review processes used.	15
	23d	Discuss implications of the results for practice, policy, and future research.	14
**OTHER INFORMATION**			
Registration and protocol	24a	Provide registration information for the review, including register name and registration number, or state that the review was not registered.	2
	24b	Indicate where the review protocol can be accessed, or state that a protocol was not prepared.	2
	24c	Describe and explain any amendments to information provided at registration or in the protocol.	2
Support	25	Describe sources of financial or non-financial support for the review, and the role of the funders or sponsors in the review.	17
Competing interests	26	Declare any competing interests of review authors.	17
Availability of data, code and other materials	27	Report which of the following are publicly available and where they can be found: template data collection forms; data extracted from included studies; data used for all analyses; analytic code; any other materials used in the review.	17

PRISMA ABSTRACT CHECKLIST

Section and Topic	Item #	Checklist item	Reported (Yes/No)
**TITLE**			
Title	1	Identify the report as a systematic review.	Yes
**BACKGROUND**			
Objectives	2	Provide an explicit statement of the main objective(s) or question(s) the review addresses.	Yes
**METHODS**			
Eligibility criteria	3	Specify the inclusion and exclusion criteria for the review.	Yes
Information sources	4	Specify the information sources (e.g. databases, registers) used to identify studies and the date when each was last searched.	Yes
Risk of bias	5	Specify the methods used to assess risk of bias in the included studies.	Yes
Synthesis of results	6	Specify the methods used to present and synthesise results.	Yes
**RESULTS**			
Included studies	7	Give the total number of included studies and participants and summarise relevant characteristics of studies.	Yes
Synthesis of results	8	Present results for main outcomes, preferably indicating the number of included studies and participants for each. If meta-analysis was done, report the summary estimate and confidence/credible interval. If comparing groups, indicate the direction of the effect (i.e. which group is favoured).	Yes
**DISCUSSION**			
Limitations of evidence	9	Provide a brief summary of the limitations of the evidence included in the review (e.g. study risk of bias, inconsistency and imprecision).	Yes
Interpretation	10	Provide a general interpretation of the results and important implications.	Yes
**OTHER**			
Funding	11	Specify the primary source of funding for the review.	Yes

Nadarajah R, *et al. Heart* 2022; 108:1020–1029. doi: 10.1136/heartjnl-2021-320036

Section and Topic	Item #	Checklist item	Reported (Yes/No)
Registration	12	Provide the register name and registration number.	Yes

REFERENCES

1. Ammenwerth E, Neyer S, Hörbst A, et al. Adult patient access to electronic health records. *Cochrane Database Syst Rev* 2021(2)
2. Poorthuis MH, Jones NR, Sherliker P, et al. Utility of risk prediction models to detect atrial fibrillation in screened participants. *European Journal of Preventive Cardiology* 2020;28(6):586-95.
3. Samuel L. Factors predisposing to the development of atrial fibrillation. *Pacing Clin Electrophysiol* 1997;20(10):2670-74.
4. Kodani E, Atarashi H. Prevalence of atrial fibrillation in Asia and the world. *Journal of Arrhythmia* 2012;28(6):330-37.
5. Haim M, Hoshen M, Reges O, et al. Prospective national study of the prevalence, incidence, management and outcome of a large contemporary cohort of patients with incident non-valvular atrial fibrillation. *J Am Heart Assoc* 2015;4(1):e001486.
6. Site HG. Clopidogrel plus aspirin versus oral anticoagulation for atrial fibrillation in the Atrial fibrillation Clopidogrel Trial with Irbesartan for prevention of Vascular Events (ACTIVE W): a randomised controlled trial. *The Lancet* 2006;367(9526):1903-12.
7. Alonso A, Krijthe BP, Aspelund T, et al. Simple risk model predicts incidence of atrial fibrillation in a racially and geographically diverse population: the CHARGE-AF consortium. *J Am Heart Assoc* 2013;2(2):e000102. doi: 10.1161/JAHA.112.000102 [published Online First: 2013/03/30]
8. Schnabel RB, Sullivan LM, Levy D, et al. Development of a risk score for atrial fibrillation (Framingham Heart Study): a community-based cohort study. *The Lancet* 2009;373(9665):739-45. doi: 10.1016/s0140-6736(09)60443-8
9. Chao T-F, Liu C-J, Lin Y-J, et al. Oral anticoagulation in very elderly patients with atrial fibrillation: a nationwide cohort study. *Circulation* 2018;138(1):37-47.
10. Snell KI, Ensor J, Debray TP, et al. Meta-analysis of prediction model performance across multiple studies: Which scale helps ensure between-study normality for the C-statistic and calibration measures? *Stat Methods Med Res* 2018;27(11):3505-22.
11. metamisc: Meta-Analysis of Diagnosis and Prognosis Research Studies [program], 2020.
12. Debray TP, Damen JA, Riley RD, et al. A framework for meta-analysis of prediction model studies with binary and time-to-event outcomes. *Stat Methods Med Res* 2019;28(9):2768-86.
13. Van Remoortel H, Scheers H, De Buck E, et al. Prediction modelling studies for medical usage rates in mass gatherings: A systematic review. *PLoS One* 2020;15(6):e0234977.
14. Peduzzi P, Concato J, Feinstein AR, et al. Importance of events per independent variable in proportional hazards regression analysis II. Accuracy and precision of regression estimates. *J Clin Epidemiol* 1995;48(12):1503-10.
15. van der Ploeg T, Austin PC, Steyerberg EW. Modern modelling techniques are data hungry: a simulation study for predicting dichotomous endpoints. *BMC Med Res Methodol* 2014;14(1):1-13.
16. Collins GS, Ogundimu EO, Altman DG. Sample size considerations for the external validation of a multivariable prognostic model: a resampling study. *Stat Med* 2016;35(2):214-26.
17. Pencina MJ, D'Agostino RB. Evaluating discrimination of risk prediction models: the C statistic. *JAMA* 2015;314(10):1063-64.

18. Hill NR, Ayoubkhani D, McEwan P, et al. Predicting atrial fibrillation in primary care using machine learning. *PLoS One* 2019;14(11):e0224582. doi: 10.1371/journal.pone.0224582 [published Online First: 2019/11/02]
